# Supplementary material for: Conditional chemoconnectomics (cCCTomics) as a strategy for efficient and conditional targeting of chemical transmission
Source: eLife. 2024 Apr 30;12:RP91927. doi: 10.7554/eLife.91927 (PMC11060718; doi:10.7554/eLife.91927)
Supplement: Figure 3—source data 3. [file elife-91927-fig3-data3.zip › Figure 3 source data3 part 2.zip!/Figure 3-Source data 3-part2/1053f3-1-CRISPResso_Report_t6djfp6u.zip!/CRISPResso_on_1053f3_3/9.Alleles_frequency_table_around_sgRNA_ggccaccagatgcaaaatcg.pdf]

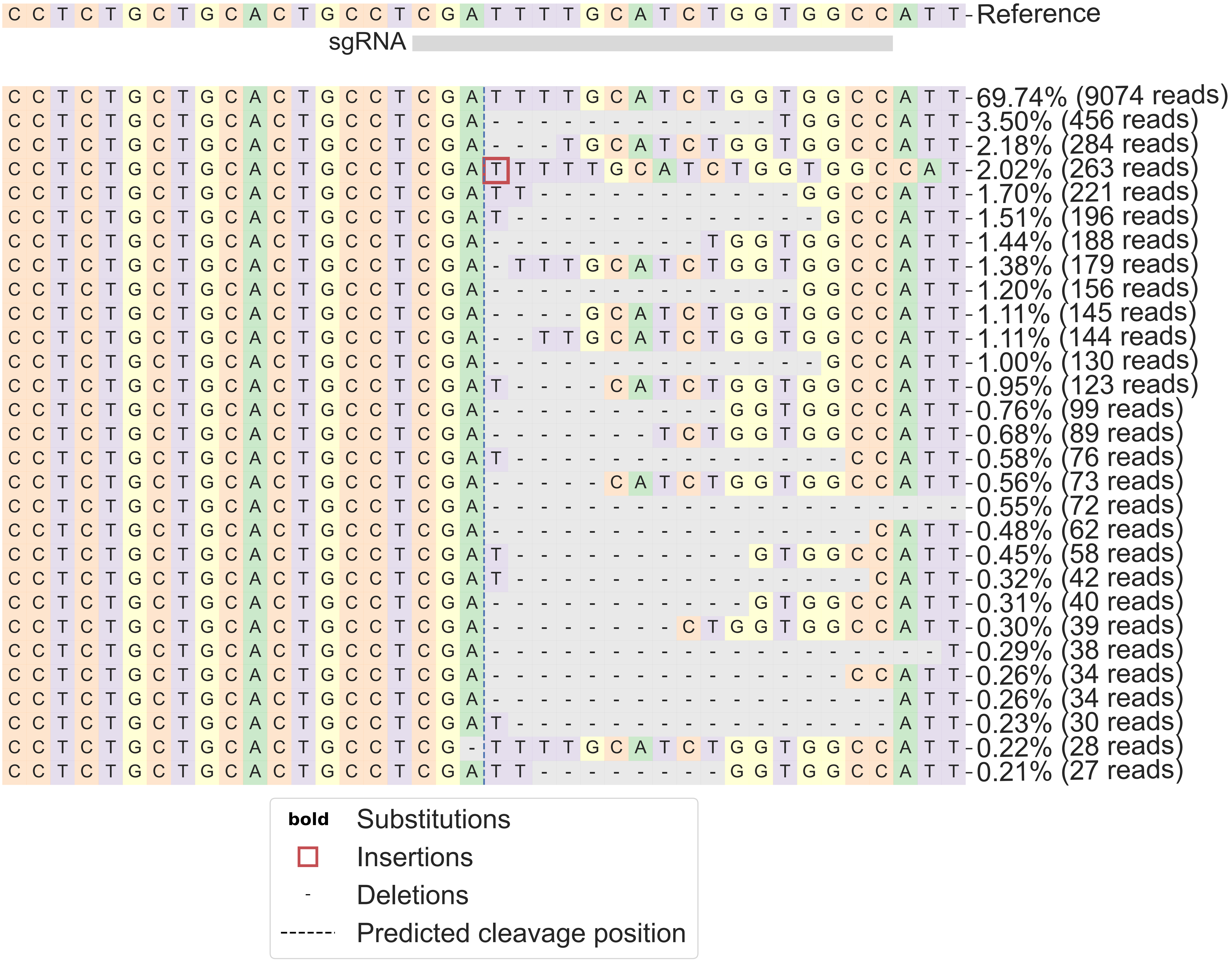

Reference
sgRNA
69.74% (9074 reads)
3.50% (456 reads)
2.18% (284 reads)
2.02% (263 reads)
1.70% (221 reads)
1.51% (196 reads)
1.44% (188 reads)
1.38% (179 reads)
1.20% (156 reads)
1.11% (145 reads)
1.11% (144 reads)
1.00% (130 reads)
0.95% (123 reads)
0.76% (99 reads)
0.68% (89 reads)
0.58% (76 reads)
0.56% (73 reads)
0.55% (72 reads)
0.48% (62 reads)
0.45% (58 reads)
0.32% (42 reads)
0.31% (40 reads)
0.30% (39 reads)
0.29% (38 reads)
0.26% (34 reads)
0.26% (34 reads)
0.23% (30 reads)
0.22% (28 reads)
0.21% (27 reads)
bold Substitutions
Insertions
Deletions
Predicted cleavage position
